# Power Defect Detection with Improved YOLOv12 and ROI Pseudo Point Cloud Visual Analytics

**DOI:** 10.3390/s26020445

**Published:** 2026-01-09

**Authors:** Minglang Xu, Jishen Peng

**Affiliations:** School of Electrical and Control Engineering, Liaoning Technical University, Huludao 125105, China; pengjishen@163.com

**Keywords:** YOLOv12, power defect detection, deep learning, ROI pseudo point cloud, visual analytics

## Abstract

**Highlights:**

**What are the main findings?**

- An improved YOLOv12 integrating PG-RA, LR-RELAN, and DSAF Loss achieves more robust power-equipment defect detection under complex backgrounds and varying illumination.
- ROI-based pseudo point cloud construction with SOR/ROR denoising provides clearer local structural patterns, improving the interpretability of defect regions beyond 2D bounding box outputs.

**What are the implications of the main findings?**

- The proposed framework offers a lightweight and deployable solution for intelligent power inspection, supporting faster and more reliable condition assessment in real-world maintenance workflows.
- The low-cost pseudo point cloud visual analytics pipeline suggests a practical path to enhance explainability in industrial defect detection.

**Abstract:**

Power-equipment fault detection is challenging in real-world inspections due to subtle defect cues and cluttered backgrounds. This paper proposes an improved YOLOv12-based framework for multi-class power defect detection. We introduce a Prior-Guided Region Attention (PG-RA) module and design a Lightweight Residual Efficient Layer Aggregation Network (LR-RELAN). In addition, we develop a Dual-Spectrum Adaptive Fusion Loss (DSAF Loss) function to jointly improve classification confidence and bounding box regression consistency, enabling more robust learning under complex scenes. To support defect-oriented visual analytics and system interpretability, the framework further constructs Region of Interest (ROI) pseudo point clouds from detection outputs and compares two denoising strategies, Statistical Outlier Removal (SOR) and Radius Outlier Removal (ROR). A Python-based graphical prototype integrates image import, defect detection, ROI pseudo point cloud construction, denoising, 3D visualization, and result archiving into a unified workflow. Experimental results demonstrate that the proposed method improves detection accuracy and robustness while maintaining real-time performance, and the ROI pseudo point cloud module provides an intuitive auxiliary view for defect-structure inspection in practical applications.

## 1. Introduction

With the rapid development of power systems and the continuous expansion of grid scale, the safe operation of transmission and distribution lines and the monitoring of equipment conditions have increasingly become critical to ensuring the stability of power systems [1]. Conventional manual inspections are characterized by high labor intensity, low efficiency, and significant safety risks. These limitations are particularly pronounced for high-voltage lines and in mountainous or otherwise complex environments, where inspection reliability is severely constrained. Consequently, automated power-defect detection methods based on computer vision and intelligent sensing technologies have gradually emerged as a major research focus [2].

In recent years, deep learning-based object detection algorithms have achieved remarkable success in industrial and computer-vision applications. Owing to their end-to-end design, single-stage architecture, and strong real-time performance, the YOLO family has been widely adopted for power-equipment defect detection [3]. On one hand, extensive efforts have been devoted to improving YOLO through lightweight network designs and enhanced multi-scale feature representation. For instance, incorporating distribution-aware localization losses can accelerate convergence and strengthen bounding box learning [4], while introducing lightweight backbones and attention mechanisms can improve feature efficiency and robustness in complex scenes [5]. In addition, depthwise separable convolution and structural redesigns have been explored to reduce computational cost while maintaining multi-scale detection capability [6]. CF-YOLO is proposed, which enhances the detection performance of multi-scale small targets in complex remote sensing scenes through improvements such as a cross-scale feature pyramid (CS-FPN) based on the feature aggregation mechanism of PAN (Path Aggregation Network), cross-scale feature aggregation, and detection head reconstruction [7]. Collectively, these developments have substantially advanced deep learning-based detection methods.

On the other hand, beyond general architectural improvements, some studies have deeply integrated the YOLO family with specific application scenarios to enhance robustness to dynamic targets, tiny objects, and complicated environments. For example, combining YOLO with spatial transformation can improve recognition under viewpoint changes [8], and adding dedicated small-object branches can boost the recall of tiny targets under cluttered backgrounds [9]. Scenario-oriented YOLO variants have also been developed for real-time detection under harsh conditions and small-object settings [10]. While these approaches improve inference efficiency and reduce false positives and missed detections to some extent, they are often evaluated in domain-specific contexts and may not explicitly address the practical requirements and constraints of power system inspection.

In electrical power system inspection, YOLO-based detectors have been actively tailored to fault identification and localization under real-world operating conditions. For example, Stefenon et al. proposed an optimized ensemble strategy combined with Weighted Boxes Fusion (WBF) to enhance insulator fault detection robustness by fusing predictions from multiple detectors [11]. In parallel, Hypertuned-YOLO and integrated EigenCAM for distribution power grid fault location were also introduced to provide interpretable evidence for the predicted fault regions, which is valuable for operational decision-making [12]. These studies, together with other YOLO variants tailored for power-grid components, demonstrate active exploration on architecture refinement, post-processing fusion, and visual interpretability in real-world inspection scenarios.

Despite the progress of YOLO-based detectors in power-equipment defect detection, current approaches still face two practical limitations. Specifically, robust identification of tiny and subtle defects in complex backgrounds remains challenging, and edge deployment calls for more effective lightweight feature aggregation and prior-aware enhancement. Moreover, defect detection is seldom coupled with ROI-guided 3D structural analysis within an integrated pipeline, and systematic ROI-level comparisons of classical denoising strategies are still scarce. To address these gaps, we are motivated to develop a unified framework that improves 2D detection performance and incorporates a practical, prototype-ready ROI-level structural visualization module to better support autonomous inspection.

Compared with two-dimensional information, three-dimensional point clouds contain rich geometric and spatial cues, which are beneficial for characterizing the structural properties of power equipment and its surrounding environment [13]. However, acquired 3D point cloud data often include substantial noise and sparse points. Without appropriate denoising, the subsequent assessment of the spatial structure of defect regions and the quality of 3D visualization can be adversely affected. Classical point cloud denoising methods, such as Statistical Outlier Removal (SOR) and Radius Outlier Removal (ROR), have been widely used to eliminate outliers and sparse points prior to downstream processing [14]. Nevertheless, existing studies either focus primarily on defect detection in 2D images without fully exploiting 3D structural cues [15], or investigate point cloud denoising in general LiDAR scenarios while paying limited attention to the characteristics of defect-level Regions of Interest (ROI) in power-inspection imagery. For instance, Gonizzi et al. [16] converts 3D point clouds into volumetric models with geometric accuracy that enables structural analysis through finite-element procedures; however, its workflow is still insufficiently integrated end-to-end with defect detection and autonomous power-operation and maintenance scenarios. In summary, there remains a lack of a unified framework and prototype system that organically integrates object detection, defect ROI-level structured representation/denoising comparisons, and 3D visualization for autonomous power operations and maintenance applications.

To address the aforementioned issues, this paper proposes a power-defect detection method based on an improved YOLOv12 and ROI pseudo point cloud visualization and analysis (YOLOv12-RPPVA). First, building upon the original YOLOv12 detection framework, we introduce a Prior-Guided Region Attention (PG-RA) module and a Lightweight Residual Efficient Layer Aggregation Network (LR-RELAN), which strengthen feature responses to critical equipment regions and improve feature utilization efficiency while maintaining low computational overhead. Furthermore, we design a Dual-Spectrum Adaptive Fusion Loss (DSAF Loss) function, which jointly enhances classification confidence and bounding box regression consistency in complex detection backgrounds, thereby improving robustness.

Building upon the improved detection module, this study constructs an ROI-level pseudo point cloud for each detected defect region and applies two representative denoising strategies—SOR and ROR—to remove and comparatively analyze outliers and sparse points. This module is intended to provide prototype-level structural visualization and interpretability for defect regions, rather than to replace LiDAR-based 3D perception. Finally, a graphical prototype system is implemented in Python, integrating image import, defect detection, ROI pseudo point cloud construction, denoising, 3D visualization, and result archiving into a unified workflow. This system provides an intuitive and convenient tool for analyzing power defects and their spatial structures.

The main contributions of this paper are summarized as follows:

(1) For multi-class power-defect detection, we develop an improved YOLOv12 model by introducing PG-RA and LR-RELAN, which enhances small-object representation while maintaining strong real-time performance.
(2) We propose DSAF, which jointly strengthens classification confidence and bounding box regression consistency, improving robustness to complex backgrounds and subtle defect patterns in real-world scenarios.
(3) We establish a defect-ROI-based pseudo point cloud visualization and denoising scheme, and systematically compare the effectiveness and applicability of SOR and ROR for ROI-level noise suppression.
(4) We implement a Python-based graphical prototype that unifies defect detection, ROI pseudo point cloud construction, denoising, 3D visualization, and result management into a single workflow, offering an intuitive tool for power-equipment condition assessment and autonomous inspection applications.

## 2. Theory/Fundamentals

### 2.1. Improved YOLOv12

**Figure 1:**
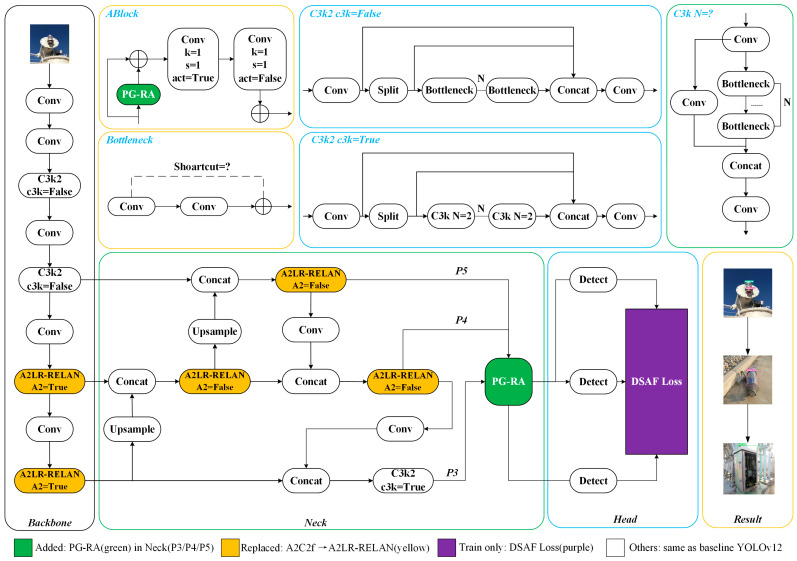
Improved YOLOv12 power defect target detection model.

The network architecture of the improved YOLOv12-based power-defect detector is shown in Figure 1.

To enhance defect-recognition accuracy and overall robustness for power-inspection tasks in complex environments, this paper performs several targeted optimizations based on the baseline YOLOv12 architecture. These improvements include incorporating PG-RA, introducing LR-RELAN, and designing DSAF Loss. These optimizations effectively enhance the model’s sensitivity to composite defects and subtle anomalies, enabling higher accuracy and stronger interference resistance in power-equipment defect detection.

To improve information processing and fine-grained representation, we jointly model a spatial Prior-Guided Region Attention module and a Lightweight Residual Efficient Layer Aggregation Network. This design strengthens feature responses in key defect regions and reduces the risk that tiny defects are ignored during detection, thereby enhancing perception and discrimination in the recognition stage. The spatial prior module adopts a channel-discrepancy activation strategy and constructs regional weight distributions by incorporating height-related distributions and statistical characteristics. In this way, the network is guided to prioritize high-incidence defect areas, such as towers and conductors, and to preserve finer details at critical spatial locations.

The residual efficient layer aggregation network combines channel grouping, 1 × 1 compression, and depthwise separable 3 × 3 convolutions. Compared with the original structure, this design markedly reduces computational cost and parameter count, making it better suited for onboard embedded platforms in power-inspection unmanned vehicles. Residual connections are retained within groups, while inter-group 1 × 1 fusion enables hierarchical feature integration. This strategy alleviates channel redundancy and increases the diversity of multi-branch features. Since the module replaces only the original convolutional units without altering input or output scales, it can be seamlessly integrated into the YOLOv12 backbone and neck. Together with PG-RA and the improved loss, these components form a complete lightweight detector for power-inspection tasks. In addition, the proposed DSAF Loss enhances feature separability, further improving the recognition of tiny defects under complex background interference and boosting overall accuracy and robustness.

### 2.2. Spatial Prior-Guided Region Attention Mechanism

In power-inspection scenarios, target objects exhibit clear spatial distribution priors. On one hand, most power facilities are concentrated in the upper half of images and around tower regions, whereas key targets rarely appear in blank areas such as the ground and sky [17]. On the other hand, many defect targets are small or slender, making them prone to being obscured by complex backgrounds [18]. Conventional channel- or spatial attention modules typically rely on the features themselves to adaptively learn salient regions without explicitly incorporating such scenario priors, which can lead to insufficient emphasis on critical areas [19].

**Figure 2:**
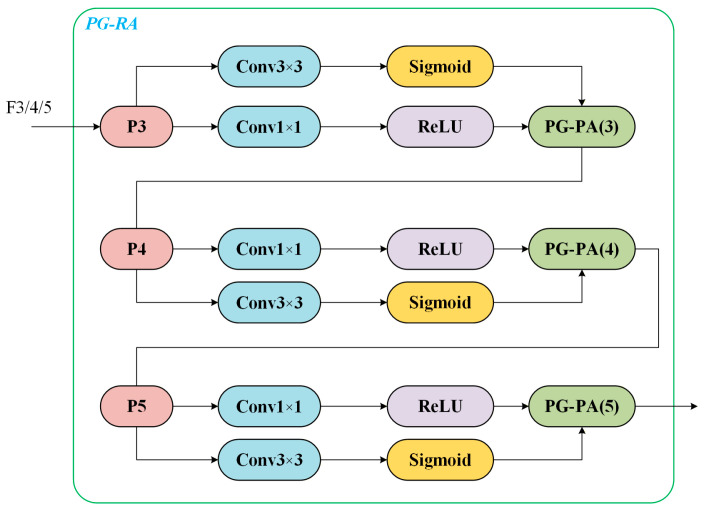
Spatial PG-RA module.

To address these issues, we introduce a spatial PG-RA module into the original YOLOv12. By constructing a spatial prior map tailored to power-inspection scenarios and jointly modeling it with network features through adaptive matching, the module guides the network to allocate greater attention during forward propagation to regions more likely to contain power equipment. This design enhances the detection of small and long-range targets, as shown in Figure 2.

Construction of the regional prior map. Let F denote the intermediate feature map (a 3D tensor) output by the l-th layer of the network

(1) $$F\in R^{C\times H\times W},$$

where C, H, and W denote the number of channels, height, and width, respectively.

Based on the statistical observation that power-inspection targets are more likely to appear at higher image locations, we construct a regional prior map using a concise and effective height-prior function $M_{prior}$:

(2) $$M_{prior}\in R^{1\times H\times W}.$$

Let the normalized height coordinate corresponding to the i-th row be denoted as $y_{i}=\frac{i}{H}$, i=1,…,H. The prior weight is then defined as

(3) $$W_{prior}\left(i,j\right)=\alpha +\left(1-\alpha \right)y_{i}^{\gamma },$$

where j=1,…,W, $\alpha \in \left(0,1\right)$ denotes the minimum prior weight, which ensures that the lower part of the image retains a certain level of response; γ>1 controls the curvature of the weight variation with respect to height. As $y_{i}$ approaches 1, the weight tends toward 1, so the upper regions receive stronger attention. When $y_{i}$ is small, the weight approaches, and the lower regions are moderately suppressed.

This construction requires no additional annotations and relies only on common-sense spatial distributions in power-inspection scenes, yielding an interpretable regional prior that provides guidance for subsequent attention learning.

To fully leverage the network’s feature-representation capability while preserving the interpretability of the prior, PG-RA does not simply multiply the prior map with the feature map. Instead, it employs a lightweight convolutional network to jointly model the features and the prior, producing an adaptive spatial attention map. First, the regional prior map is expanded along the batch dimension and concatenated with the feature map along the channel dimension to form $F_{c}$:

(4) $$F_{c}=Concat\left(F,W_{prior}\right)\in R^{\left(C+1\right)\times H\times W}.$$

Here, the batch dimension refers to the first dimension N of the feature tensor, i.e., $F_{N}\in R^{N\times C\times H\times W}$, where N is the number of input images processed in parallel in one forward pass. Therefore, “along the batch dimension” means replicating the prior map $M_{prior}$ for N times to obtain $M_{prior}^{\left(N\right)}\in R^{N\times 1\times H\times W}$, so that each sample in the batch is associated with the same spatial prior.

Subsequently, we adopt a lightweight two-layer design consisting of a 1 × 1 convolution for channel reduction followed by a 3 × 3 convolution for spatial-context modeling to extract spatial attention features. Here, the symbol ‘+’ in the previous description was only a shorthand for sequential stacking (i.e., ‘followed by’), rather than an element-wise summation. The spatial attention feature is represented as

(5) $$\tilde{F}=\delta \left({Conv}_{1\times 1}\left(F_{c}\right)\right),$$

(6) $$A=\sigma \left({Conv}_{3\times 3}\left(\tilde{F}\right)\right),$$

where ${Conv}_{1\times 1}$ is used for channel compression to reduce computational cost, while ${Conv}_{3\times 3}$ is employed to introduce local spatial context. $\delta \left(\cdot \right)$ denotes the ReLU activation function, and $\sigma \left(\cdot \right)$ denotes the Sigmoid function that normalizes the attention values to $\left[0,1\right]$. The resulting spatial attention map is $A\in R^{1\times H\times W}$, thus simultaneously encodes both the regional prior and the feature-response information.

Within the overall detection framework, we embed the PG-RA module into the neck of YOLOv12 to perform region-guided enhancement of multi-scale features. Specifically, three-scale feature maps from the FPN/PAN structure, namely $P_{3}$, $P_{4}$, and $P_{5}$, correspond to small-scale, medium-scale, and large-scale targets, respectively. Each scale is processed by an independent PG-RA module in the following sequence: $\overset{\wedge }{P_{3}}=PG-RA\left(P_{3}\right)$, $\overset{\wedge }{P_{4}}=PG-RA\left(P_{4}\right)$, $\overset{\wedge }{P_{5}}=PG-RA\left(P_{5}\right)$. The enhanced feature maps $\overset{\wedge }{P_{3}}$, $\overset{\wedge }{P_{4}}$, and $\overset{\wedge }{P_{5}}$ are then fed into the decoupled detection head for classification and regression. This design not only leverages the regional prior to emphasize elevated equipment and small targets, but also preserves rich semantic representations for medium-scale and large-scale objects, leading to a notable improvement in multi-scale detection performance in power-inspection scenarios.

### 2.3. Lightweight Residual Efficient Layer Aggregation

**Figure 3:**
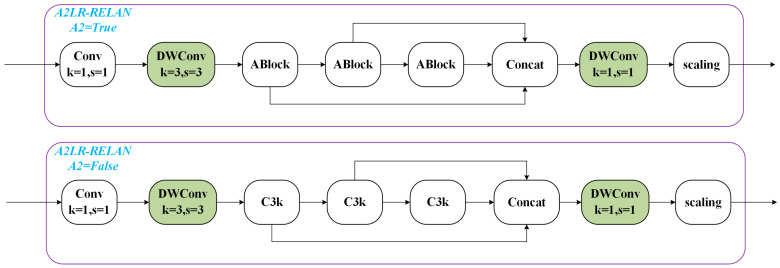
Lightweight Residual Efficient Layer Aggregation Network.

Structures such as A2C2f, which are commonly used in the backbone and neck of YOLOv12, achieve strong feature representation through “multi-branch + multi-level aggregation.” However, they also introduce two issues. First, the computational and memory costs are high: at a high resolution of 1280 × 1280, standard 3 × 3 convolutions with large channel widths incur substantially increased FLOPs, which is unfavorable for deployment on edge devices with limited computing resources, such as power-inspection unmanned vehicles [20]. Second, feature redundancy may arise: features generated by convolutions in adjacent layers are often highly correlated, and naive stacking can lead to channel redundancy and reduced network efficiency [21]. To address these issues, we replace A2C2f with a Lightweight Residual Efficient Layer Aggregation Network (LR-RELAN). The goal is to markedly reduce computation with minimal loss of accuracy and to improve the utilization efficiency of effective features through carefully designed residual connections and group-wise aggregation, as shown in Figure 3.

Naming clarification: The proposed LR-RELAN is an ELAN-style (Efficient Layer Aggregation Network) lightweight variant that further introduces residual connections and group-wise/depthwise operations for efficiency. Therefore, it can be regarded as a Lightweight Residual ELAN (LR-ELAN)-type design, but we use the term LR-RELAN throughout this paper to explicitly emphasize the residual-enhanced lightweight aggregation mechanism.

The core idea can be summarized as follows: channel grouping + lightweight intra-group convolution (DWConv 1 × 1) + intra-group residual connections + inter-group aggregation. With fewer convolutions, this design aims to achieve representational capability superior to the original A2C2f.

Let the input features be $X\in R^{C\times H\times W}$. First, divide the channel dimension into groups. The number of channels per group is $\frac{C}{G}$ (assuming it can be divided evenly): $X=\left[X^{\left(1\right)},X^{\left(2\right)},\ldots ,X^{\left(G\right)}\right]$, $X^{\left(g\right)}\in R^{\frac{C}{G}\times H\times W}$.

For each group $X^{\left(g\right)}$, a small block utilizing “1 × 1 channel compression + depthwise separable 3 × 3 convolution + 1 × 1 channel restoration + residual” is employed for local feature extraction:

Step 1: Channel compression:

(7) $$U^{\left(g\right)}=\phi_{1}\left({Conv}_{1\times 1}\left(X^{\left(g\right)}\right)\right),$$

where $U^{\left(g\right)}\in R^{C_{m}\times H\times W}$, $C_{m}=\frac{C}{G\cdot r}$ (r>1) is the channel compression ratio, $\phi_{1}$ is BN + ReLU.

Step 2: Depthwise separable convolution (lightweight):

(8) $$V^{\left(g\right)}=\phi_{2}\left({DWConv}_{3\times 3}\left(U^{\left(g\right)}\right)\right),$$

where DWConv represents depthwise separable convolution, where each channel is a separate convolution, significantly reducing the FLOPs (Floating-point Operations Per Second) index.

Step 3: Channel restoration: Restore the output channel to $\frac{C}{G}$:

(9) $${\tilde{X}}^{\left(g\right)}={Conv}_{1\times 1}\left(V^{\left(g\right)}\right).$$

Step 4: Intra-group residual connection: $Y^{\left(g\right)}=X^{\left(g\right)}+{\tilde{X}}^{\left(g\right)}$. Finally, concatenate the outputs of all groups along the channel dimension, and then apply a 1 × 1 convolution for inter-layer/group aggregation and channel rearrangement. Z represents the output feature of LR-RELAN:

(10) $$Y=Concat\left(Y^{\left(1\right)},\ldots ,Y^{\left(G\right)}\right)\in R^{C\times H\times W},$$

(11) $$Z=\phi_{3}\left({Conv}_{1\times 1}\left(Y\right)\right),$$

where $\phi_{1}\left(\cdot \right)$, $\phi_{2}\left(\cdot \right)$, and $\phi_{3}\left(\cdot \right)$ denote the same nonlinear transform BN + ReLU. The subscripts (1,2,3) are used only to indicate that the corresponding layers have independent learnable parameters (different BN statistics and affine parameters).

Compared to a standard convolutional layer with the same number of input channels C and a convolution kernel size of 3 × 3, the FLOPs of standard convolution are approximately

(12) $${FLOPs}_{conv}\propto C^{2}\cdot K^{2}\cdot H\cdot W.$$

The FLOPs of the DWConv + 1 × 1 combination in each group of LR-RELAN are approximately

(13) $${FLOPs}_{group}\propto C_{m}\cdot K^{2}+C_{m}^{2}\ll C^{2},$$

where for $C_{m}\cdot K^{2}$ is DWConv, due to $C_{m}=\frac{C}{G\cdot r}$ and G,r>1, the overall FLOPs can be reduced to about 30% to 60% of the original C2f/ELAN (specifically related to hyperparameters). At the same time, multiple sets of residuals and 1 × 1 aggregation ensure feature diversity and expressive ability. Here, ‘about 30% to 60%’ denotes the relative computational cost ratio,

(14) $$\eta =\frac{\mathrm{FLOPs}\left(\mathrm{LR}-\mathrm{RELAN}\right)}{\mathrm{FLOPs}\left(C2f/\mathrm{ELAN}\right)},$$

which forms a range because it depends on the key hyperparameters of LR-RELAN, including the number of channel groups g and the channel compression ratio α. In typical settings (e.g., $g\in \left[2,4\right]$ and $\alpha \in \left[0.5,0.75\right]$), η generally falls within 0.3–0.6, indicating that LR-RELAN requires roughly 30–60% of the FLOPs of the original block.

It can be seen that grouping + channel compression + depthwise separable convolution can significantly reduce computational complexity, which is the key reason for the light weight of the module.

### 2.4. Dual-Spectrum Adaptive Fusion Loss

In practice, power-equipment defects often exhibit hybrid characteristics: some are dominated by appearance cues (texture, color, or semantic patterns), whereas others rely more heavily on structural or boundary-sensitive cues. The conventional YOLO training objective combines classification, objectness, and regression terms, which may not fully capture confidence estimation and localization quality for such heterogeneous defect patterns [22]. To further enhance robustness in complex power-defect detection scenarios, we propose DSAF Loss.

In complex power-inspection scenarios, the EIoU Loss adopted in the baseline model handles bounding box center offset, aspect ratio, and scale variation in different ways. This inconsistency can lead to uneven convergence behavior and fluctuating training stability [23], which may result in insufficient localization accuracy and consequently undermine the reliability of defect assessment for power equipment. When the goal is to simultaneously improve localization accuracy for small targets, enhance robustness to imbalanced sample distributions, and ensure consistency between the regression branch and confidence prediction, it is also essential to keep the training process as stable as possible and to avoid increasing computational complexity during inference.

Multi-label classification loss (CLS) is a loss function for multi-label classification in the form of cross-entropy, used to distinguish specific categories of targets, such as electrical towers, insulators, lines, obstacles, etc. The definition of classification loss is

(15) $${Loss}_{cls}=-\sum_{c=1}^{C}\left[y_{c}\log({\hat{p}}_{c})+\left(1-y_{c}\right)\log\left(1-{\hat{p}}_{c}\right)\right],$$

where C is the total number of categories; $y_{c}$ is the true category label; and ${\hat{p}}_{c}$ is the predicted category probability.

Object Confidence Loss (OBJ): A confidence score is predicted at each grid point or feature anchor point to determine whether an object exists within a candidate box. The definition of Object Confidence Loss is

(16) $${Loss}_{obj}=\sum_{i=0}^{B^{2}}\sum_{j=0}^{S}H\left[-\log\left(p\right)+BCE\left(n,\hat{n}\right)\right],$$

(17) $$BCE\left(n,\hat{n}\right)=-\left[n\log\left(\hat{n}\right)+\left(1-n\right)\log\left(1-\hat{n}\right)\right],$$

(18) $$H=I_{i,j}^{obj},$$

where B represents the number of target detection grids, and S represents the number of candidate boxes corresponding to each grid. H is the weight and I is the probability that the j-th anchor box in the i-th grid contains a target, while n and $\hat{n}$ represent the confidence of the true box and the predicted box, respectively. When n=1, it represents that there is a target at that position; when n=0, it represents the background.

For complex scenarios in power inspection, the Inner-SIoU loss term [24] is introduced. SIoU further incorporates angle constraints and shape constraints on the basis of the traditional IoU series (such as GIoU, DIoU, CIoU), enabling the model to converge faster and achieve more stable box matching during the localization process. It can also introduce angle and shape constraints while considering the overlapping area, thereby improving localization consistency. They are defined as

(19) $${Loss}_{Inner-SIoU}=1+\frac{\left(\Delta +\Omega \right)}{2}-{IoU}^{inner},$$

(20) $${IoU}^{inner}=\frac{inter}{union},$$

where Δ is an angle constraint; Ω is a shape constraint; inter is the size of the input box; and union is the size of the output box.

Bounding box localization loss (BOX): A slenderness constraint term is added to the Inner-SIoU loss term, defined as

(21) $${Loss}_{box}={Loss}_{Inner-SIoU}+C_{elong},$$

(22) $$C_{elong}=\gamma_{e}\left(r-1\right)\left(1-e^{-\frac{\left|\Delta \omega \right|+\left|\Delta h\right|}{\omega_{g}+h_{g}}}\right),$$

where $C_{elong}$ is a slender constraint term, $\gamma_{e}$ is a parameter for adjusting the imbalance between positive and negative samples, and $\omega_{g}$, $h_{g}$ represent the width and height of the detection box, where $\omega_{g}$ and $h_{g}$ denote the width and height of the ground-truth (GT) bounding box, respectively (and $\omega_{p}$, $h_{p}$ denote the width and height of the predicted box, if used).

Focal Loss: It can mitigate the dominant effect of easily classifiable samples on the loss, thereby focusing on difficult-to-classify targets. Its definition is

(23) $$FocalLoss=-\alpha_{t}{\left(1-p_{t}\right)}^{\gamma }\log\left(p_{t}\right),$$

where t denotes a unified timestamp generated when processing an input image. This timestamp is used to uniquely group and name the saved outputs (e.g., ROI pseudo point clouds and denoised results) from the same inference run. $\alpha_{t}$ is the parameter for adjusting the difficulty of samples, γ is the parameter for adjusting the imbalance between positive and negative samples, $p_{t}$ represents whether the samples are easy to distinguish, and ${\left(1-p_{t}\right)}^{\gamma }$ indicates that the contribution of easy-to-distinguish samples can be reduced.

Distribution Focal Loss (DFL): It is an improvement of the loss function used for bounding box coordinate regression. DFL is not a loss function for classification, but rather for improving the accuracy of bounding box coordinate regression, allowing the model to learn a regression value that follows a discrete distribution. By making the model output a distribution instead of a direct numerical value, it is more refined and smoother than traditional L1/L2 regression. The definition of DFL is as follows:

(24) $${Loss}_{dfl}=-\sum_{i=1}^{N}p_{i}^{*}\log{\hat{p}}_{i},$$

where ${\hat{p}}_{i}$ represents the predicted distribution probability, while $p_{i}^{*}$ denotes the actual distribution probability generated based on the floating-point value of the coordinate.

In summary, after introducing the Inner-SIoU loss and Distribution Focal Loss functions, the DSAF Loss function is ultimately defined as

(25) $${Loss}_{DSAF}=\lambda_{cls}{Loss}_{cls}+\lambda_{obj}{Loss}_{obj}+\lambda_{box}{Loss}_{box}+\lambda_{dfl}{Loss}_{dfl},$$

where the corresponding weight coefficients are set to $\lambda_{cls}=0.5$, $\lambda_{obj}=1.0$, $\lambda_{box}=7.5$, and $\lambda_{dfl}=1.5$ (kept fixed for all epochs unless otherwise stated). These values follow common YOLO loss balancing and were further verified by a small grid search on the validation split.

DSAF aims to improve the consistency between confidence estimation and localization by introducing stronger geometry-aware and fine-grained regression constraints. Specifically, the regression branch incorporates an Inner-SIoU term to stabilize alignment related to shape and orientation, and adopts the DFL to refine boundary modeling via distribution-based regression. By balancing these components with appropriate weights, DSAF encourages the model to produce more reliable confidence scores and more accurate bounding boxes, thereby reducing missed detections and false alarms in cluttered backgrounds.

## 3. Materials and Methods

**Figure 4:**
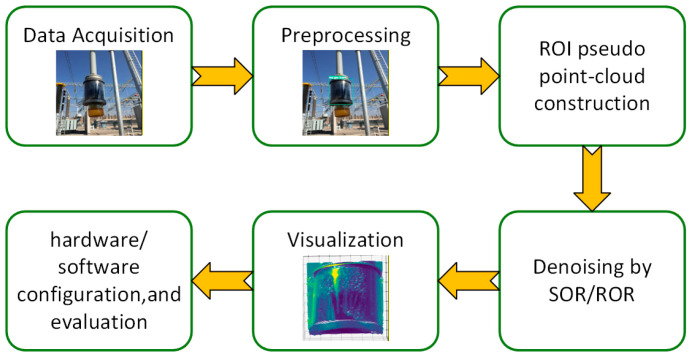
The simulated flowchart.

The entire process, from data acquisition, preprocessing, ROI pseudo point cloud construction, denoising, and visualization to final software and hardware configuration and evaluation, is illustrated in the flowchart shown in Figure 4.

### 3.1. Data Acquisition

The training environment for the proposed algorithm is built on Windows 11. The hardware configuration includes an AMD Ryzen 9 7950X3D processor (Advanced Micro Devices, Inc., Santa Clara, CA, USA), an NVIDIA GeForce RTX 4070 Super GPU, and 64 GB of RAM. The implementation is based on Python 3.12, and PyCharm 2025.1 is used as the integrated development environment (IDE).

The improved YOLOv12-based detection algorithm imposes high requirements on the dataset quality. To achieve the expected training performance, we construct a dataset consisting of on-site images collected from transmission-line inspections conducted by a power supply company. The dataset contains 6601 images with a total of 8753 annotated instances. We randomly split the dataset into training/validation/test sets with a ratio of 18:5, resulting in 5186/1415 images, respectively. The split is performed at the image level to avoid information leakage across sets. All models were trained for 100 epochs with a batch size of 16. We use SGD with momentum 0.937 and weight decay 5 × 10^−4^. The initial learning rate is set to 0.01 and scheduled by cosine annealing with a 3-epoch warmup. The input image size is 1280 × 1280 (unless otherwise stated). The dataset is annotated using LabelImg and includes labels for 17 different types of power-equipment defects, as listed in Table 1.

We did not adopt k-fold cross-validation. Considering the dataset size and the heavy training cost of YOLO-style detectors, we used a fixed hold-out split. To reduce randomness, we set a fixed random seed and repeated training three times; the reported metrics are the average results.

### 3.2. Pseudo Point Cloud Visualization System

To validate the proposed power-defect detection and point cloud processing methods, we develop a Python-based graphical prototype system. The system is implemented using PyCharm as the development environment for the interface and integrates the improved YOLOv12 detector, ROI pseudo point cloud construction, pseudo point cloud denoising, and 3D visualization. It provides an end-to-end workflow that covers image import, defect detection, pseudo point cloud generation, and result saving.

#### 3.2.1. Object Detection and Category Visualization

The system first encapsulates a YOLO detector module (YOLO Detector). Based on the Ultralytics implementation of YOLOv12, it loads the trained weights and automatically selects the running device (GPU/CPU). After initialization, the model reads the class names and generates a set of highly distinguishable pseudo-color mappings according to the number of categories. These colors are used to render bounding boxes for different defect types, enabling intuitive visualization of multi-class defect recognition results on the interface.

During operation, the user uploads a power-inspection image through the interface. The system passes the image path to the YOLO detector for forward inference, obtaining the bounding box coordinates, class IDs, and confidence scores for each defect target. The system then draws rectangular bounding boxes on the original image using category-specific colors and overlays text labels of class names. The resulting annotated image is displayed in real time on the left side of the interface and is simultaneously saved to a local results directory as a timestamped JPG file, facilitating record keeping and subsequent experimental comparisons.

#### 3.2.2. Construction of Defect ROI Pseudo Point Clouds

After obtaining reliable 2D defect localization, we further introduce ROI pseudo point clouds as a prototype-level structural visualization layer to enhance the interpretability of defect-region presentation. To construct local 3D information from visual detection results, the system designs a Point Cloud Extractor module that generates pseudo 3D point clouds for each detected ROI. Specifically, for each bounding box $\left[x_{1},y_{1},x_{2},y_{2}\right]$ output by YOLOv12, the corresponding ROI patch is first cropped from the original image, and a 2D grid coordinate set $\left(x,y\right)$ with the same size as the ROI is generated. The ROI is then converted to grayscale, and the normalized pixel intensity is used as the “depth” dimension z. These three components are combined to form a 3D coordinate set $\left(x,y,z\right)$, thereby constructing a pseudo point cloud that reflects the spatial distribution and intensity variations in the defect region. This process is implemented by the extract_from_roi function and returned as a NumPy 2.2.0 array.

Although this point cloud is not sampled by real LiDAR but is a local pseudo 3D representation constructed from image coordinates and grayscale mapping, it is valuable in the prototype-validation stage for comparing spatial structures and intensity distributions across different regions. This enables intuitive visualization of defect characteristics in the “spatial-intensity” domain.

#### 3.2.3. Comparison of Denoising for Defective ROI Pseudo Point Cloud

To emulate common denoising workflows in practical point cloud processing, the system implements a Point Cloud Denoiser module that provides two representative methods—Statistical Outlier Removal (SOR) and Radius Outlier Removal (ROR). Their key differences mainly lie in decision criteria, algorithmic principles, and applicable scenarios [25], as shown in Table 2.

SOR: For each point, utilize a KD-Tree to query its nearest neighbors and compute the average distance distribution of the neighborhood. Based on the global mean and standard deviation, mark points with neighborhood average distances greater than μ+std_ratio·σ as outliers and remove them, while retaining the main dense regions, thereby eliminating isolated noise points and local clusters of outliers [26].

ROR: using a given search radius as the threshold, this method counts the number of neighbors for each point within the radius. If the neighbor count is lower than a predefined minimum, the point is considered to lie in a sparse region and is removed. This approach is effective in suppressing noise in point clouds with non-uniform density [27].

When the point cloud density is uneven, such as LiDAR scenarios, SOR is generally more stable; when the density is relatively uniform, such as object scanning, ROR tends to be more intuitive and straightforward. For initial processing when parameter settings are uncertain, it is advisable to prioritize statistical filtering as the first denoising option.

The system firstly generates an original pseudo point cloud for each detected ROI. It then applies the selected filtering strategy to obtain the denoised pseudo point cloud and reports the number of retained points and the denoising rate (the proportion of removed points), enabling quantitative evaluation of the noise-suppression capability of different filters. This article compares SOR and ROR on defective ROI pseudo point clouds, aiming to evaluate the applicability of classical filtering strategies in the structured expression of defect-level ROI, and provide a reference for visual verification and parameter selection in unmanned operation and maintenance prototype systems.

#### 3.2.4. Three-Dimensional Visualization and Result Management

To facilitate intuitive analysis of point cloud processing results, the system encapsulates an Mpl Canvas class based on Matplotlib’s 3D plotting functions (Matplotlib 3.7.1). On the right side of the interface, it provides a side-by-side comparison of the original and denoised pseudo point clouds for each detected target. Each target corresponds to a grouped panel that displays two 3D scatter plots in parallel. The pseudo point clouds are rendered using $\left(x,y,z\right)$ coordinates. The viridis colormap is used to encode intensity values as colors, accompanied by a color bar to interpret depth variations along the z-axis. Users can rotate the viewpoint by dragging the mouse, enabling convenient inspection of structural details from different perspectives.

In addition, the system saves NumPy files for both the original and denoised data for each detected target. The filenames include a unified timestamp, target index, and class name, allowing for rapid loading in standalone scripts for statistical analysis or comparative algorithmic experiments.

Overall, without altering the core algorithmic structure, the prototype links visual detection, ROI pseudo point cloud construction, denoising, 3D visualization, and data archiving into an integrated pipeline. This visualization design is intended to enable intuitive verification of local structures and intensity variations within defect ROIs and to provide interpretable evidence for engineering selection between SOR and ROR. Consequently, it offers an intuitive and reproducible experimental platform and an engineering-oriented validation environment for power-defect detection methods.

#### 3.2.5. Graphical Prototype System and Asynchronous Mechanism

The system implements the main window and interactive layout using a Python-based GUI framework. The left panel displays the input image and detection results, while the right panel presents side-by-side views of the original and denoised ROI pseudo point clouds. To prevent the inference process from blocking the user interface, a dedicated detection thread is introduced. Progress and results are returned via a signal–slot mechanism, enabling asynchronous inference and smooth interaction.

In a nutshell, without altering the core algorithmic structure of the proposed method, the prototype links visual detection, ROI pseudo point cloud construction, pseudo point cloud denoising, 3D visualization, and data archiving into an integrated workflow. It provides an intuitive and reproducible experimental platform as well as an engineering-oriented validation environment for the improved YOLOv12 and pseudo point cloud-based power-defect detection method.

## 4. Results

### 4.1. Evaluation Metrics

To verify whether the training achieves the expected performance, we adopt Precision (P), Confidence (C), Recall (R), Average Precision (AP), and mean Average Precision (mAP) as evaluation metrics for the object detection algorithm. Precision measures the proportion of true positives among all samples predicted as positive by the model. Recall reflects the proportion of all real positive samples that are successfully detected by the model. They are defined as

(26) $$P=\frac{TP}{TP+FP},$$

(27) $$R=\frac{TP}{TP+FN},$$

where TP denotes the number of true positives samples correctly predicted as positive; FP denotes the number of false positives samples that are actually negative but are mistakenly predicted as positive; and FN denotes the number of false negatives samples that are actually positive but are missed by the model and predicted as negative.

For real-time performance evaluation, we adopt detection speed as the primary indicator, measured by the number of image frames that the model can process per unit time (Frames Per Second, FPS).

AP is defined as the area under the P–R curve, where P is plotted on the y-axis and R on the x-axis. mAP is computed by averaging AP over all categories and is used to measure the overall detection performance of the algorithm. They are defined as follows:

(28) $$AP=\int_{0}^{1}P\left(R\right)dR,$$

(29) $$mAP=\frac{\sum_{k=1}^{n}\int_{0}^{1}P\left(R\right)dR}{n}.$$

**Figure 5:**
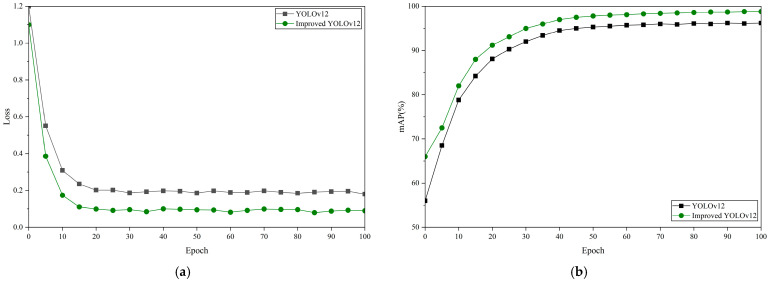
Variation curve. (**a**) Total loss; (**b**) mAP variation curve.

To evaluate the performance gains brought by the proposed improvements, we plot the total loss and mAP curves of the baseline and the optimized models during training, before and after the different model enhancements, as shown in Figure 5.

As shown in Figure 5, the improved YOLOv12 exhibits faster and smoother convergence than the baseline, with a steeper early loss reduction and stable low loss after mid-training.

**Figure 6:**
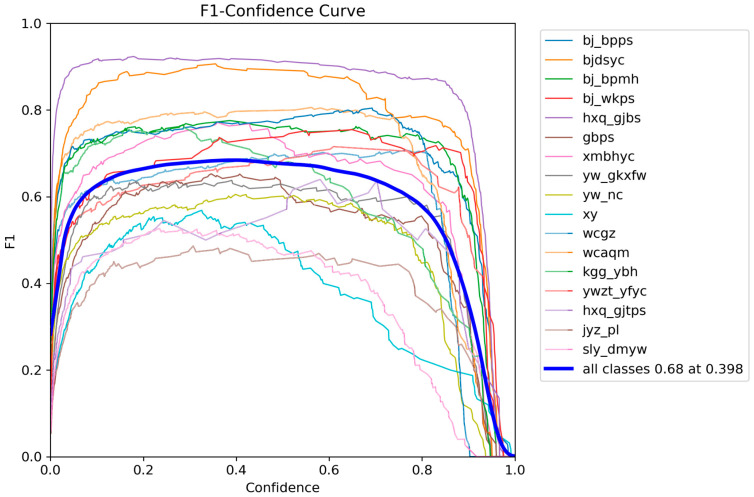
F1-Confidence curve for different defects.

**Figure 7:**
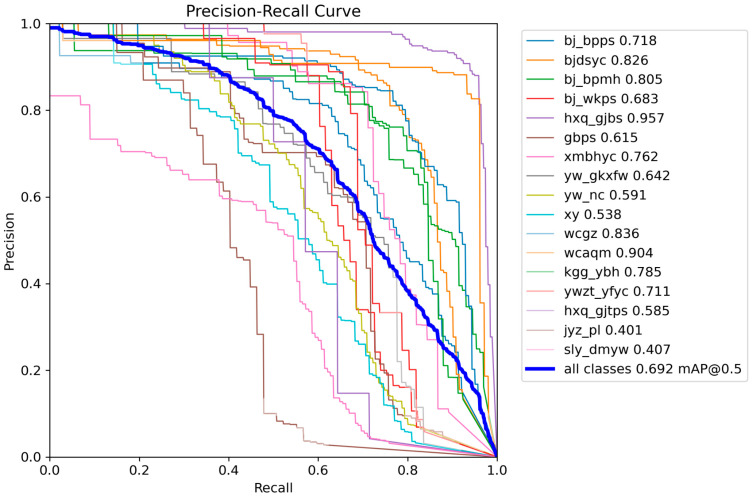
Precision–Recall curve for different defects.

**Figure 8:**
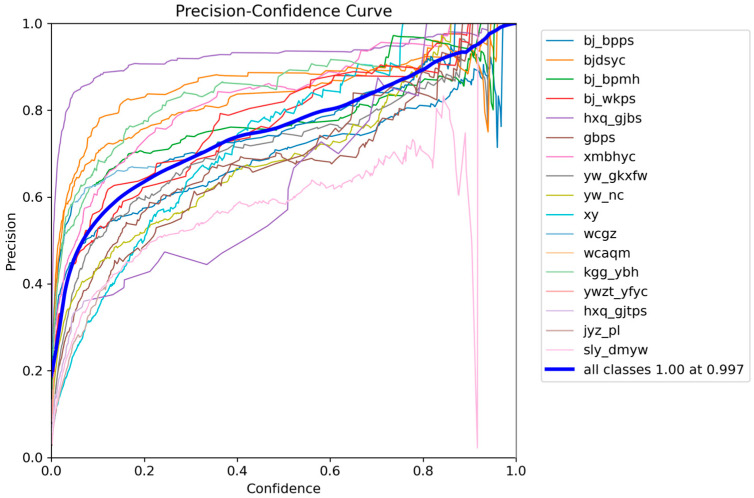
Precision–Confidence curve for different defects.

**Figure 9:**
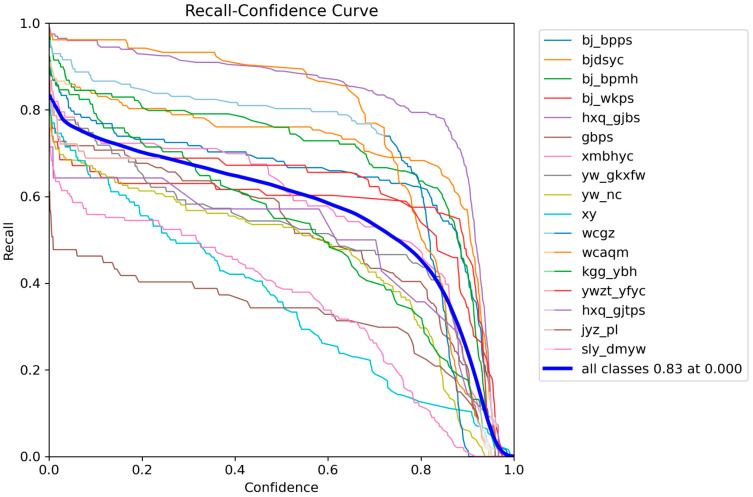
Recall–Confidence curve for different defects.

And it further indicates a rapid improvement in mAP during the first stage of training, followed by a high and stable plateau, demonstrating enhanced learning efficiency and detection capability. These results suggest that the proposed improvements yield robust optimization behavior and strong class-discriminative performance under complex defect distributions, providing a solid basis for practical deployment. We further present per-class F1-Precision–Confidence–Recall relationship curves to examine category-wise learning dynamics, as shown in Figure 6, Figure 7, Figure 8 and Figure 9.

They provide a category-wise view of the Confidence-dependent detection behavior. In Figure 6, the averaged F1 curve over all classes peaks at approximately 0.68 when the confidence threshold is around 0.40, indicating that a threshold in the range of 0.35–0.45 yields a near-optimal Precision–Recall trade-off in our setting. Several classes achieve high F1 peaks (≈0.8–0.9), suggesting stable separability, whereas classes with lower and more fluctuating peaks likely suffer from limited samples or inter-class confusion.

Figure 7 reflects the expected Precision–Recall trade-off: Precision remains relatively high at moderate recall levels but drops sharply as recall approaches 1, implying that near-complete coverage would substantially increase false positives.

Figure 8 and Figure 9 further show that Precision increases while Recall decreases with higher Confidence thresholds. Overall, raising the threshold produces more conservative predictions with fewer false alarms but more missed detections, whereas lowering it reduces misses at the cost of increased false alarms.

### 4.2. Comparative Experiments

We design comparative experiments involving six different network models, including FVI-DDNet [28], Mask R-CNN [29], RTPV-YOLO [30], YOLOv8 [31], YOLOv12 [32], and our improved method, YOLOv12-RPPVA. These models are evaluated on the constructed dataset, and the AP values for 17 categories of power-inspection defect targets are reported. The results are presented in Table 3.

Meanwhile, to comprehensively assess the overall performance of different models in the defect-detection task, we set the confidence threshold of all models to 40% and compare five metrics—Precision, Confidence, Recall, mAP, and detection speed. The evaluation results are summarized in Table 4. FPS denotes Frames Per Second, i.e., the number of input images processed per second during inference. It reflects the inference throughput rather than the number of detected faults per second.

As summarized in Table 3 and Table 4, YOLOv12-RPPVA achieves the best overall performance across the 17 defect categories. Compared with the baseline YOLOv12, it delivers consistent AP improvements for representative classes, such as meter-related and respirator/insulator defects, indicating that the dual-spectrum loss and structural enhancements effectively strengthen discriminative learning. Traditional two-stage methods: FVI-DDNet and Mask R-CNN show weaker Precision, Recall and mAP trade-offs and fall short of real-time requirements, while RTPV-YOLO benefits from multimodal fusion but remains limited in Recall and speed. In contrast, YOLOv12-RPPVA improves Precision and mAP to 98.9% and 96.8% and raises Recall to 93.2% with only a minor FPS drop to 42.6, demonstrating a well-balanced accuracy–efficiency profile and strong potential for practical power-inspection deployment.

### 4.3. Ablation Experiments

**Figure 10:**
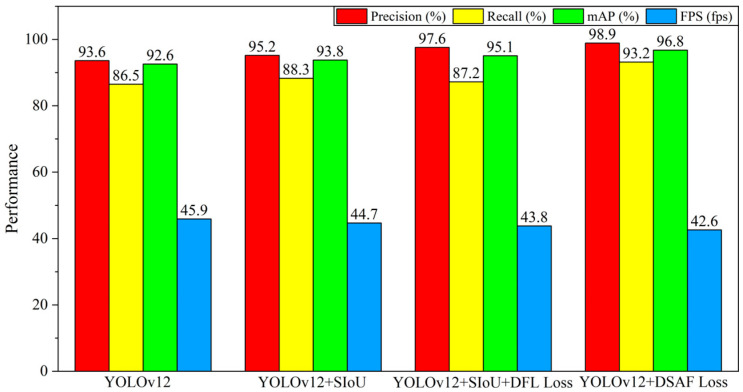
Ablation experiment results graph.

To further clarify the performance contributions of each improvement to YOLOv12 in the power-inspection defect-detection task, we conduct ablation experiments. Starting from the baseline YOLOv12, we progressively add SIoU, DFL, and the final DSAF Loss module to form multiple network configurations, which are then evaluated on the constructed test dataset. We then objectively analyze the comprehensive performance of each configuration using Precision, Recall, mAP, and detection speed to quantify the practical gains brought by these strategies. The results are presented in Table 5 and Figure 10.

As summarized in Table 5 and Figure 10, the baseline YOLOv12 already achieves strong performance on power-equipment defect detection. Introducing SIoU yields consistent gains in Precision, Recall, and mAP with only a negligible FPS drop, indicating improved geometric alignment with minimal cost to real-time inference. Adding DFL further enhances boundary modeling and accuracy but may reduce recall, whereas the full DSAF Loss restores and boosts Recall while maintaining high precision/mAP and stable speed. Overall, these results confirm that the proposed loss design strategy provides a favorable tradeoff between accuracy, robustness, and efficiency in complex power detection scenarios.

**Figure 11:**
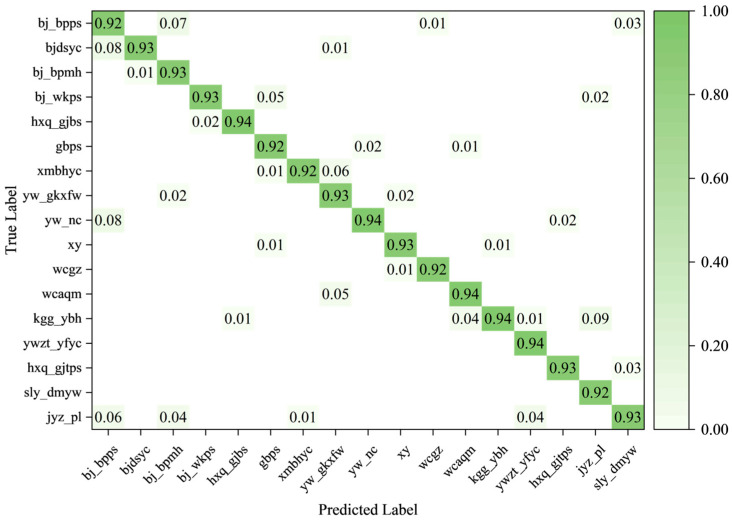
Confusion matrix of YOLOv12.

**Figure 12:**
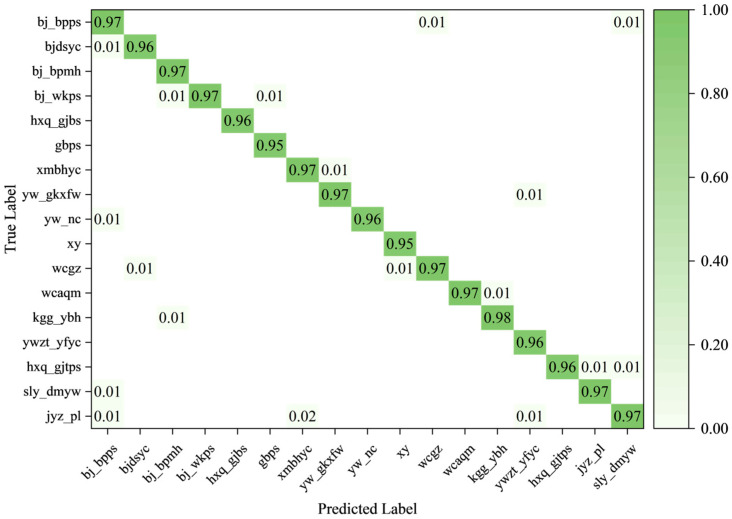
Confusion matrix of YOLOv12-RPPVA.

The classification confusion matrices of YOLOv12 and the improved model are presented in Figure 11 and Figure 12, respectively, illustrating the prediction performance for each category of power-equipment defects.

In these matrices, rows correspond to ground-truth classes, and columns correspond to predicted classes. Blank entries indicate values of zero. The values on the main diagonal represent the proportion of correctly classified samples for each defect category; values closer to 1 indicate more accurate predictions for that class. In contrast, off-diagonal values reflect misclassification. A larger number of off-diagonal entries or higher values indicates more frequent false classifications. In summary, the improved model demonstrates noticeably higher classification accuracy than YOLOv12 and a lower misclassification rate.

### 4.4. Comparative Results of Denoising for Defect ROI Pseudo Point Clouds

**Figure 13:**
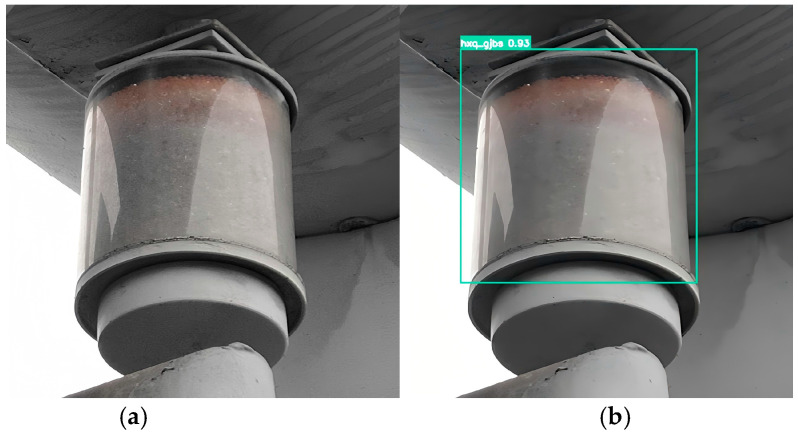
Detection image. (**a**) Original image. (**b**) Labeled image.

**Figure 14:**
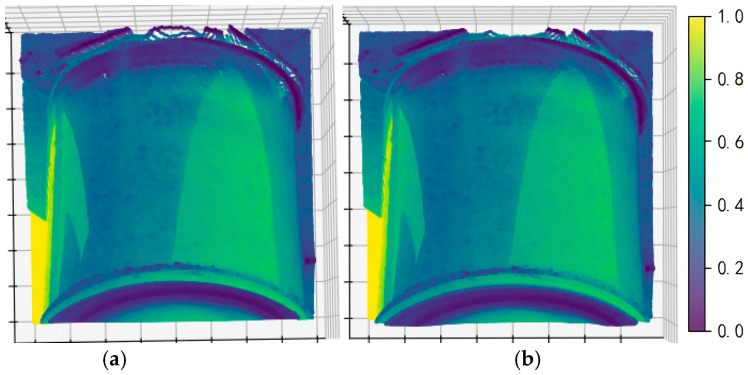
SOR denoising image. (**a**) Original pseudo point cloud. (**b**) Pseudo point cloud denoised by SOR.

To improve the readability and engineering interpretability of structural representations in defect regions, the implementation was developed in Python, with Py-Charm 2025.1 used as the development environment. Based on the collected dataset, we construct ROI-level pseudo point clouds for detected defect regions and conduct a comparative analysis using two classical strategies: SOR and ROR. This comparison aims to evaluate the suitability of different filtering strategies for defect-ROI-level structured representation and to provide references for point cloud visualization and assisted interpretation in autonomous power-inspection prototype systems, as shown in Figure 13 and Figure 14.

As shown in Figure 13 and Figure 14, the pseudo point cloud processed using SOR remains highly consistent with the original point cloud in terms of overall structure, target contours, and local density distribution, without obvious loss of principal points or morphological damage. This observation indicates that, under the current defect-ROI representation and data conditions, SOR can suppress random outliers at a relatively low cost while preserving the main defect structure. Therefore, it is more suitable as a robust preprocessing choice for ROI-level structural visualization.

**Figure 15:**
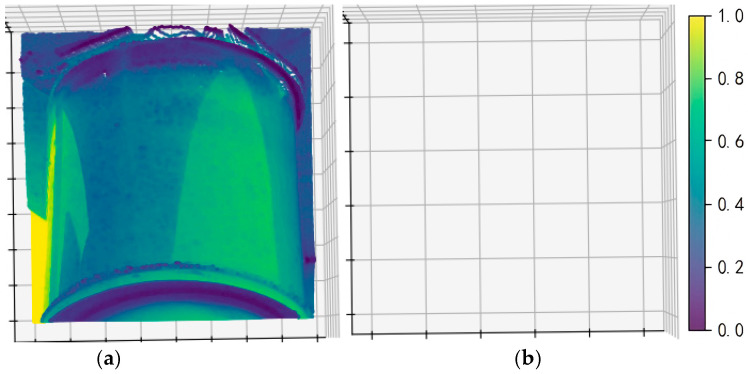
ROR denoising image. (**a**) Original pseudo point cloud. (**b**) Pseudo point cloud denoised by ROR; it becomes nearly empty.

In contrast, Figure 15 shows that ROR leads to substantial point reduction or even complete point cloud loss under our experimental setting. This is mainly because, affected by long-range sampling and variations in equipment morphology, the ROI point density in power-inspection scenarios is often non-uniform, whereas ROR is highly sensitive to density uniformity and parameter selection.

Figure 15b appears to contain only grids because, under our current experimental setting, ROR removes almost all points in the defect-ROI pseudo point cloud due to non-uniform point density; therefore, no scatter points remain to be displayed and only the 3D coordinate grid/axes are visible. Consequently, within the current pseudo point cloud validation framework for power-inspection defect ROIs, ROR exhibits insufficient stability and should not be prioritized. SOR better meets the robustness requirements of practical scenarios.

### 4.5. ROI Pseudo Point Cloud-Assisted Visualization Analysis

**Figure 16:**
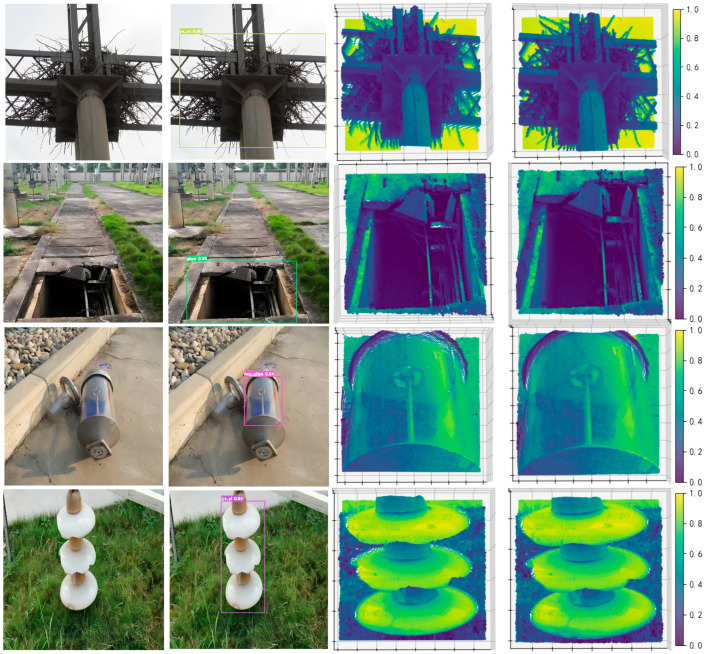

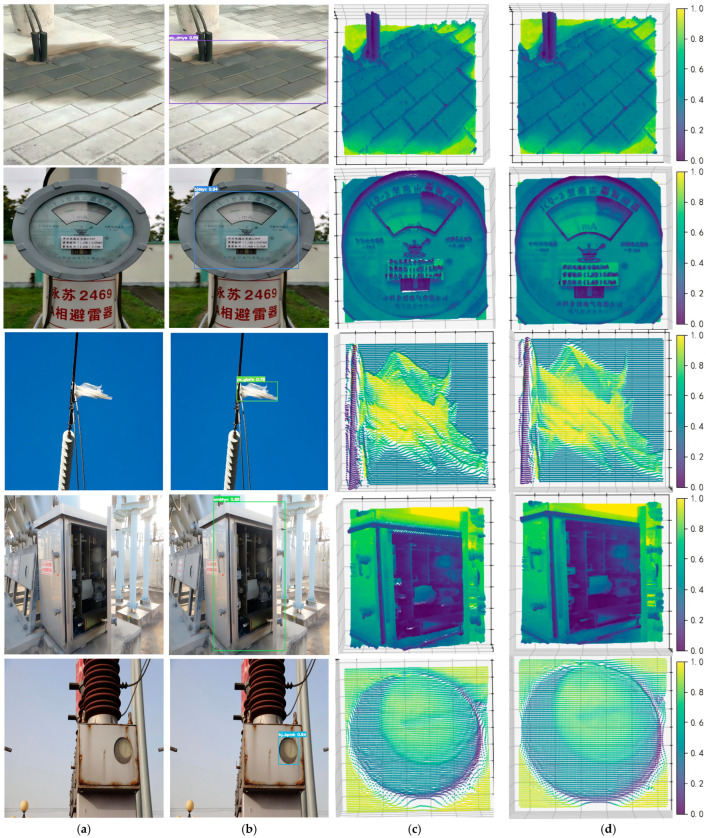
Experimental results for different types of defects. (**a**) Original images; (**b**) detection annotation images; (**c**) original ROI pseudo point clouds; (**d**) SOR-denoised ROI pseudo point clouds.

We select nine representative defect categories, including foreign objects: a bird nest (yw_nc), cover plate damage (gbps), respirator silicone damage (hxq_gjtps), insulator cracking (jyz_pl), oil leakage with ground contamination (sly_dmyw), an incorrect meter reading (bjdsyc), suspended debris (yw_gkxfw), abnormal closure of control cabinet door (xmbhyc), and a blurred meter dial (bj_bpmh). Figure 16 presents comparisons of “original image–detection annotation–original ROI pseudo point cloud–SOR-denoised ROI pseudo point cloud,” enabling an intuitive assessment of detection performance for different defect categories under complex scenarios.

As observed in Figure 16, different defect types exhibit markedly different degrees of reliance on pseudo point cloud assistance. For categories dominated by color changes, texture details, or semantic cues—such as the incorrect meter reading, blurred meter dial, silicone discoloration, and ground oil contamination—the pseudo point cloud provides limited direct evidence for discrimination. Its primary value lies in offering auxiliary visualization of defect-region localization and environmental structural context, while the final recognition still mainly relies on the visual branch of the improved YOLOv12.

In contrast, foreign-object defects such as suspended hanging debris and bird nests exhibit prominent volumetric and spatial-occupancy characteristics. Abnormal closure of control cabinet doors, switchgear state anomalies, and certain large-scale shell damage or insulator cracking can also lead to noticeable changes in equipment geometry or pose. For these defect types, the SOR-denoised ROI pseudo point clouds clearly reveal local structural distributions, providing engineers with an intuitive auxiliary perspective for rapid verification and status assessment.

Therefore, within the overall detection framework, pseudo point cloud information mainly serves two roles: providing strong auxiliary, and in some cases dominant, cues for structural or deformation-related defects, and imposing spatial constraints to suppress false alarms for texture-dominated defects. On one hand, it markedly improves the detection rate and robustness for defects with prominent geometric characteristics, such as foreign objects, bird nests, and abnormal cabinet-door closure. On the other hand, by enforcing spatial consistency of candidate regions, it reduces the false-alarm probability of visually detected meter-related defects and oil-leakage cases under complex backgrounds, thereby enabling more comprehensive and reliable perception across diverse power-defect types.

To summarize, the ROI pseudo point cloud module in our framework is positioned primarily for prototype-level interpretable visualization and structural-assisted defect analysis. Together with the high-accuracy results of the improved YOLOv12, it forms a unified workflow and a systematic validation pathway for autonomous power operation and maintenance. These findings further indicate that our approach is centered on the enhanced visual detection capability of the improved YOLOv12, while ROI pseudo point clouds and the SOR/ROR comparison provide complementary support for structural interpretation and engineering-oriented system implementation.

## 5. Discussion

Motivated by the engineering requirements of power-equipment defect detection and grounded in prior studies on visual detection and 3D/pseudo-3D structural representation, this work proposes an integrated solution that combines an improved YOLOv12 with ROI pseudo point cloud denoising and visualization. Compared with conventional approaches that rely solely on visual cues, our results show that introducing structural enhancement modules such as PG-RA and LR-RELAN, together with the proposed DSAF Loss for joint optimization of localization and category learning, improves detection accuracy and robustness under complex illumination, background interference, and target-scale variations. This finding is consistent with prior conclusions that attention mechanisms enhance salient defect-region representation and that multi-scale feature aggregation mitigates missed detections of small and fine-grained defects. Building on these insights, our study further demonstrates—within the high-noise, high-reflection, and frequently occluded conditions of power inspection—that structural lightweighting can be achieved without sacrificing precise representation. This suggests that, for industrially deployable detection systems, network design should not focus exclusively on parameter reduction or peak accuracy, but should instead pursue a more interpretable balance among real-time performance, stability, and maintainability.

From the perspective of the working hypotheses, this study implicitly rests on two key premises. First, power-equipment defects exhibit local texture and morphological cues in the image domain that can be stably captured, enabling more reliable bounding box regression and category discrimination through an improved detection network. Second, although ROI-constructed pseudo point clouds are not equivalent to real 3D sensor sampling, they can provide additional structural cues for defect regions via an approximate “intensity–space” mapping, thereby enhancing intuitive understanding of defect spatial distribution and deformation-related characteristics. The experimental results partially support these hypotheses. The improved detection performance suggests the effectiveness of the enhanced feature representation and loss design, while the reduced noise and improved structural clarity in visualization indicate that classical denoising ideas such as SOR/ROR still have engineering value in the pseudo point cloud setting. Nevertheless, it should be emphasized that the “depth” in pseudo point clouds is derived from normalized grayscale/intensity values and is inherently influenced by illumination, material reflectance, and imaging parameters. As a result, its geometric meaning is only weakly constrained physically; under strong specular reflections, shadow occlusions, or surface contamination, intensity variations may deviate from true structural changes. Accordingly, the pseudo point cloud visualization in this work should be interpreted primarily as an aid for qualitative inspection and structural cues, rather than as a basis for rigorous 3D measurement or quantitative deformation estimation.

Placing the significance of this study in a broader context, our work offers insights aligned with the emerging trend of shifting industrial visual inspection from “black-box” decision-making toward interpretable assistance. On one hand, the stringent safety and reliability requirements of power-equipment inspection demand that algorithms not only improve accuracy but also reduce missed detections and false alarms. By constructing low-cost pseudo point clouds on top of detection results and performing denoising-based visualization, our approach provides maintenance personnel with more intuitive spatial cues of defect regions, which can help shorten the time required for manual verification and secondary interpretation.

On the other hand, in scenarios where real 3D sensors are costly or difficult to deploy, the pseudo point cloud strategy offers a feasible “software compensating for hardware” pathway. By leveraging existing visual acquisition systems along with lightweight structural enhancements and post-processing, the system can deliver inspection outputs that are both real-time and interpretable. This idea also has potential for transfer to other industrial inspection domains, such as substation appearance inspection, utility-tunnel structural anomaly identification, and detection of surface corrosion or damage on equipment.

Future research can be extended along three directions. First, at the data level, incorporating real LiDAR, depth point clouds, or multi-view structural information would help validate the generalizability of the current ROI-level structural analysis and establish a more rigorous benchmark for 3D defect representation. Second, at the methodological level, it is promising to explore defect-morphology-oriented, ROI-aware learned denoising and lightly coupled multimodal consistency mechanisms, enabling 3D information to evolve from “interpretable visualization” toward “quantifiable performance gains.” Third, for practical deployment, cross-scenario generalization and reliability evaluation should be strengthened. We recommend building larger-scale benchmarks that cover diverse equipment types, seasonal illumination, weather conditions, and camera parameters, and incorporating uncertainty estimation and active learning to assess the method’s support for safety-critical tasks in a manner closer to real operation and maintenance workflows.

## 6. Conclusions

To address the challenges of frequent missed detections and false alarms across multiple defect types, as well as the difficulty of intuitively presenting defect spatial structures in autonomous power inspection, this paper proposes a power-defect detection method based on an improved YOLOv12 with pseudo point cloud denoising and visualization. At the 2D visual-detection level, considering the characteristics of small targets, diverse categories, and complex background interference, we introduce the spatial prior-guided PG-RA module and the lightweight LR-RELAN structure, together with the proposed DSAF Loss. These designs strengthen responses to critical regions and improve feature-expression efficiency, enabling a better balance among accuracy, robustness, and real-time performance.

At the 3D information-processing level, we construct defect ROI-level pseudo point clouds based on detected bounding boxes as a low-cost, prototype-level means for structural visualization and assisted interpretation. This approach characterizes local “spatial–intensity” differences within defect regions, and we conduct a targeted comparison of two representative denoising strategies, SOR and ROR. Experimental results show that statistical filtering provides more stable noise suppression under the non-uniform density conditions typical of power-inspection point clouds and effectively improves the readability of defect-region spatial structures. In contrast, radius-based filtering is more susceptible to density non-uniformity in this task, limiting its applicability. It should be noted that this pseudo point cloud module is intended to enhance engineering readability and system completeness, and is not equivalent to a 3D detection branch based on real LiDAR or depth sensors.

Furthermore, we implement a graphical prototype system that integrates image import, defect detection, ROI point cloud construction, denoising, and 3D visualization, providing an engineering-oriented validation platform for rapid analysis and reproducible evaluation of multi-class power defects.

In conclusion, this paper takes the high-accuracy, real-time detection capability of the improved YOLOv12 as its primary contribution, while the ROI pseudo point cloud module and the comparison between SOR and ROR provide complementary support for engineering interpretability and system-level validation. The proposed approach not only verifies the effectiveness of the improved YOLOv12 in power-inspection scenarios but also explores the practical value of point cloud denoising for structural analysis within defect regions. By bridging real-world inspection requirements and engineering implementation constraints, this work offers a balanced solution that jointly considers accuracy, real-time performance, and interpretability, providing a reproducible technical reference and a systematic pathway for the continued evolution of autonomous O&M in smart grids.

## Figures and Tables

**Table 1:** Different defect categories in power inspection.

Defect Category	Abbreviation	Number of Images	Number of Annotations
The meter dial is damaged	bj_bpps	605	630
The meter reading is incorrect	bjdsyc	519	645
The meter dial is blurry	bj_bpmh	635	665
The meter casing is damaged	bj_wkps	339	339
Respirator: silicone discoloration	hxq_gjbs	878	908
The cover plate is damaged	gbps	478	543
The door of the control box is not closing properly	xmbhyc	302	311
Foreign object: suspended object hanging in the air	yw_gkxfw	456	492
Foreign object: bird nest	yw_nc	634	672
smoking	xy	560	578
Not wearing work attire	wcgz	624	738
Not wearing a helmet	wcaqm	439	521
The switch cabinet has been closed	kgg_ybh	54	372
Abnormal oil level of the oil seal in the breather	ywzt_yfyc	248	250
Respirator: silicone body damaged	hxq_gjtps	75	89
Insulator rupture	jyz_pl	309	324
Oil leakage: ground oil contamination	sly_dmyw	589	676
Total		6601	8753

**Table 2:** Comparison between SOR and ROR.

Comparison Item	SOR	ROR
Judgment Basis	Global Statistical Characteristics (Mean, Standard Deviation)	Local Neighborhood Density Parameter
Parameter	K (number of neighbors) + α (multiple of standard deviation)	r (radius) + minimum number of neighbors
Sensitivity to density changes	Insensitive, adaptable to non-uniform density	Sensitive, requiring relatively uniform density
Suitable for point cloud types	LiDAR point cloud, outdoor environment, significant density variations	Industrial scanning, clear structure, uniform density
Feature	Robust and highly adaptable	Simple, direct, and fast
Typical usage	Remove distant noise points and isolated points	Eliminate local sparse points and isolated edge points

**Table 3:** AP values of different methods on power defects.

Abbreviation	FVI-DDNet	Mask R-CNN	RTPV-YOLO	YOLOv10	YOLOv12	YOLOv12-RPPVA
bj_bpps	78.2	86.4	88.2	90.1	92.3	96.7
bjdsyc	78.7	84.4	88.4	89.9	92.6	95.9
bj_bpmh	78.4	87.1	87.2	90.2	93.1	96.8
bj_wkps	78.5	85.6	88.7	90.8	92.9	97.0
hxq_gjbs	77.9	84.3	88.6	90.3	93.5	96.3
gbps	78.4	84.2	88.1	90.4	92.1	94.7
xmbhyc	78.2	84.7	88.9	90.5	92.2	97.2
yw_gkxfw	78.9	83.9	89.1	90.7	92.6	96.9
yw_nc	78.5	84.6	87.8	90.8	93.5	96.3
xy	77.9	84.4	87.6	89.9	93.4	95.3
wcgz	79.1	84.3	87.2	89.8	92.1	96.7
wcaqm	78.6	84.5	88.5	90.7	93.8	96.5
kgg_ybh	78.7	83.5	88.2	91.2	94.0	97.6
ywzt_yfyc	78.3	86.1	87.2	90.9	93.7	95.9
hxq_gjtps	78.2	83.9	87.5	91.5	92.6	96.3
jyz_pl	77.8	82.9	87.9	91.2	92.3	96.5
sly_dmyw	77.9	84.1	87.6	89.6	93.4	96.9

**Table 4:** Comparison of comprehensive performance of different methods on power defects.

Methods	Precision (%)	Recall rate (%)	mAP (%)	FPS (fps)
FVI-DDNet	81.7	95.6	78.5	20.7
Mask R-CNN	84.3	91.0	82.0	19.6
RTPV-YOLO	87.9	88.8	86.5	28.2
YOLOv10	91.6	85.4	88.9	40.7
YOLOv12	93.6	86.5	92.6	45.9
YOLOv12-RPPVA	98.9	93.2	96.8	42.6

**Table 5:** Ablation experiment results.

Methods	Precision (%)	Recall Rate (%)	mAP (%)	FPS (fps)
YOLOv12	93.6	86.5	92.6	45.9
YOLOv12 + SIoU	95.2	88.3	93.8	44.7
YOLOv12 + SIoU + DFL	97.6	87.2	95.1	43.8
YOLOv12 + DSAF Loss	98.9	93.2	96.8	42.6

## Data Availability

The datasets presented in this article are not readily available due to confidentiality and security restrictions.

## Abbreviations

The following abbreviations are used in this manuscript:

YOLO	You Only Look Once
ROI	Region of Interest
PG-RA	Prior-Guided Region Attention
LR-RELAN	Lightweight Residual Efficient Layer Aggregation Network
DSAF	Dual-Spectrum Adaptive Fusion
SOR	Statistical Outlier Removal
ROR	Radius Outlier Removal
IDE	Integrated Development Environment
AP	Average Precision
mAP	Mean Average Precision
O&M	Operations and Maintenance
